# Poorer sustained attention in bipolar I than bipolar II disorder

**DOI:** 10.1186/1744-859X-9-8

**Published:** 2010-02-15

**Authors:** Chian-Huei Kung, Sheng-Yu Lee, Yun-Hsuan Chang, Jo Yung-Wei Wu, Shiou-Lan Chen, Shih-Heng Chen, Chun-Hsien Chu, I-Hui Lee, Tzung-Lieh Yeh, Yen-Kuang Yang, Ru-Band Lu

**Affiliations:** 1 Institute of Behavioral Medicine, College of Medicine, National Cheng Kung University, Tainan, Taiwan, Republic of China; 2 Department of Psychiatry, College of Medicine, National Cheng Kung University, Tainan, Taiwan, Republic of China; 3 Department of Psychiatry, National Cheng Kung University Hospital, Tainan, Taiwan, Republic of China; 4 Institute of Allied Health Sciences, College of Medicine, National Cheng Kung University, Tainan, Taiwan, Republic of China

## Abstract

**Background:**

Nearly all information processing during cognitive processing takes place during periods of sustained attention. Sustained attention deficit is among the most commonly reported impairments in bipolar disorder (BP). The majority of previous studies have only focused on bipolar I disorder (BP I), owing to underdiagnosis or misdiagnosis of bipolar II disorder (BP II). With the refinement of the bipolar spectrum paradigm, the goal of this study was to compare the sustained attention of interepisode patients with BP I to those with BP II.

**Methods:**

In all, 51 interepisode BP patients (22 with BP I and 29 with BP II) and 20 healthy controls participated in this study. The severity of psychiatric symptoms was assessed by the 17-item Hamilton Depression Rating Scale and the Young Mania Rating Scale. All participants undertook Conners' Continuous Performance Test II (CPT-II) to evaluate sustained attention.

**Results:**

After controlling for the severity of symptoms, age and years of education, BP I patients had a significantly longer reaction times (F_(2,68) _= 7.648, *P *= 0.001), worse detectability (*d'*) values (F_(2,68) _= 6.313, *P *= 0.003) and more commission errors (F_(2,68) _= 6.182, *P *= 0.004) than BP II patients and healthy controls. BP II patients and controls scored significantly higher than BP I patients for *d' *(F = 6.313, *P *= 0.003). No significant difference was found among the three groups in omission errors and no significant correlations were observed between CPT-II performance and clinical characteristics in the three groups.

**Conclusions:**

These findings suggested that impairments in sustained attention might be more representative of BP I than BP II after controlling for the severity of symptoms, age, years of education and reaction time on the attentional test. A longitudinal follow-up study design with a larger sample size might be needed to provide more information on chronological sustained attention deficit in BP patients, and to illustrate clearer differentiations between the three groups.

## Introduction

The prevalence of bipolar disorder (BP) is estimated at 3.5% to 6.4% of the general population [1,2], and 30% to 50% of those in remission will not achieve premorbid psychosocial function levels [3]. Accordingly, evidence has shown that poor functional outcome is highly associated with cognitive impairment, and may persist through the remission period [4].

However, most previous studies only focused on type I bipolar disorder (BP I) with regard to neuropsychological aspects, mainly because type II bipolar disorder (BP II) was often underdiagnosed or misdiagnosed [5]. Recently, a new bipolar spectrum paradigm has begun to appear in the research literature and in clinical practice [6]. The distinctions between BP I and BP II have been reported in several studies, which indicate that BP I and BP II are in different diagnostic categories with regard to genetic [7,8], biological [9], clinical [10,11] and pharmacological [12] aspects. Therefore, studies that examine the differences between BP I and BP II should be given greater attention.

Previous studies have reported that BP I patients may have cognitive function impairment, and the magnitude of cognitive dysfunction was greater than that of patients with BP II, even in the remittance phase [13]. However, some studies have reported that BP II patients performed significantly worse than BP I patients on multiple measures of cognitive function [14,15]. The discrepancy of these studies may be attributed to the inclusion of patients with various levels of disease severity. Summers *et al*. [14] did not control for the mood state of the patients in their study; in particular, manic symptoms were not assessed. In the study of Harkavy-Friedman *et al*. [15], the recruited BP participants consisted of suicide attempters experiencing depressive episodes; this may have been why their results contradicted other findings [13,16,17]. We therefore suspect that mood symptoms might account for the underperformance on cognitive tests among BP patients.

Sustained attention is a basic requirement for information processing. Nearly all aspects of cognitive processing, such as encoding, storage, planning and problem solving, take place during periods of sustained attention [18,19]. Individuals with sustained attention deficits may be unable to adapt to environmental demands or modify behaviours, including the inhibition of inappropriate behaviour [20]. Accordingly, sustained attention deficit was among the most commonly reported impairments in BP patients, even for those in remission [21-24]. Therefore, sustained attention deficit may be enduring and may represent a stable characteristic trait rather than a temporary state in BP patients [22,25]. Investigators have inferred that sustained attention deficit might not be secondary to an acute clinical state, but rather may constitute a vulnerability marker in the process of BP [26]. In addition, Clark *et al*. [27] suggested that sustained attention deficit may also account for cognitive impairment in other domains [27]. Sustained attention can be quantified through neuropsychological assessments using continuous performance tests (CPTs). Various studies have reported a decrease in target sensitivity during various CPT task performances among euthymic BP patients. Bora *et al*. [28] enrolled 71 BP patients (37 manic patients and 34 euthymic patients) and 34 healthy controls to illustrate that impaired target detection and reaction time inconsistencies seemed to represent trait-related impairments of BP, and that manic patients had increased commission errors and vigilance deficits. When assessing a patient's attention, CPT-II results may be affected by the possible deleterious effects of disease course, duration of illness and the number of mood episodes [26,28]. In accord with Bora *et al*.'s [28] study, which indicated that sustained attention and attentional impulsivity might be affected by mood states, BP patients who were recruited in the present study were screened to exclude those who currently had mood episodes.

To our knowledge, few reports have focused on the differences between patients with BP I and BP II with respect to sustained attention. Such a relationship may further our understanding of sustained attention between the two bipolar subgroups. The goal of this study is to compare the sustained attention of interepisode patients with BP I or BP II disorder.

## Methods

The present study was conducted at National Cheng Kung University Hospital, Tainan, Taiwan, and was approved by the Institutional Review Board for the Protection of Human Subjects. Written informed consent was obtained from each participant before inclusion into the study.

### Participants

A total of 51 BP patients (22 with BP I and 29 with BP II) were recruited from the psychiatric outpatient facility of the National Cheng Kung University Hospital. Each participant was first interviewed by an attending psychiatrist for an initial evaluation and then interviewed by a well trained research team member, using the Diagnostic and Statistical Manual of Mental Disorders, fourth edition (DSM-IV) and the validated modified Chinese version of the Modified Schedule of Affective Disorder and Schizophrenia - Lifetime (SADS-L), a semistructured interview based upon DSM-IV criteria to verify the diagnosis [29-31].

All patients for whom the clinical diagnosis could not be verified by SADS-L were excluded from the study. The diagnosis of BP was made according to DSM-IV, except for BP II, where the 4-day hypomania duration was replaced by a 2-day criterion. A large number of empirical data have validated the 2-day duration to be a more adequate criterion [2,32]. Exclusion criteria included the presence of any other DSM-IV axis I diagnosis, concomitant medical illness, neurological disorder and/or brain organic conditions, and past history of diagnosis of illegal substance and alcohol use disorders.

Patients who scored lower than 10 on the 17-item Hamilton Depression Rating Scale (HDRS)[33] and the Young Mania Rating Scale (YMRS)[34] for more than 2 weeks were considered to be in a euthymic state. In this study, however, all patients had been in a remission state for 1 week or more before they participated in the study; therefore, we defined all patients as in the interepisode stage. Clinical variables were collected, such as diagnosis, illness duration, and symptom ratings.

Additionally, 20 healthy volunteers were recruited as controls among acquaintances in the community. They were screened through the SADS-L to exclude participants with prior psychiatric history. Exclusion criteria for the controls were significant mental illness, neurological disorders, alcohol and drug abuse, and a history of major mental disorder among first-degree relatives.

### Symptom and neuropsychological assessment

#### Diagnostic and symptom measurements

The SADS-L is a semistructured interview aimed at formulating the main diagnoses based upon DSM-IV criteria with good inter-rater reliability [29,31]. The 17-item HDRS is used for assessing the severity of depression and has gained considerable acceptance within the international community, including Taiwan [35]; it is probably the most widely used rating scale for depression in both practice and research settings. In the present study, clinical raters assessed the presence of symptoms described in the HDRS over the past week. The YMRS is an 11-item instrument in which a rater ranks symptoms of mania on 5 explicitly defined grades of severity. The YMRS yields a score ranging from 0 to 60, with higher scores representing greater psychopathology. The YMRS is a credible assessment of manic symptoms and is deemed acceptable within the international community and Taiwan [36]. In the present study, clinical raters assessed the presence of symptoms described in the YMRS over the past week.

#### Conners' Continuous Performance Test (CPT-II)

The CPT-II lasts for several minutes to assess the maintenance of focused attention. Optimal performance requires an adequate level of arousal, combined with an element of executive control to resist distraction and inhibit responses to stimuli resembling targets [27].

Respondents are required to press the space bar on a computer keyboard when any letter other than "X" appears. The interstimulus intervals are 1, 2 and 4 s, with a display time of 250 ms [37]. Overall, it takes approximately 14 min to complete the task and all participants were given practice tasks prior to the actual administration of the test. Some variables of sustained attention measured by CPT-II are described below.

CPT-II produces a standard set of performance measures, which include the number of errors of omission and errors of commission. Errors of omission occur when the participant fails to respond to the target stimulus, whereas errors of commission occur when the participant responds to a non-target (X) stimulus. Hit reaction time (hit RT) represents the mean response time (ms) for all target responses over the full six trial blocks. Hit reaction time standard error (HRT SE) represents the consistency of response times and expresses the standard error response to targets. The detectability (*d'*) provides information on how well the examinee discriminates between targets and non-targets.

According to Lachman's [38] trade-off effect, significant correlations among hit RT, *d' *and errors suggests the occurrence of a trade-off between speed and accuracy. Therefore, multivariate analysis of covariance (MANCOVA) was used to control for the hit RT in order to compare the CPT-II performance among the three groups.

### Statistical analysis

χ^2 ^analyses were used to test the difference in gender distribution. The comparisons of age, years of education, illness duration and clinical symptoms (HDRS and YMRS scores) were analyzed through multivariate analysis of variance (MANOVA). The Pearson correlation test was used to test the associations between clinical variables, demographic variables and CPT-II performance. Finally, we conducted MANCOVA with hit RT, age, years of education and symptoms rating scores as covariates to compare the CPT-II performance among BP I patients, BP II patients and healthy controls. All analyses were performed using SPSS V.13.0 for Windows (SPSS, Chicago, IL, USA).

## Results

### Clinical and demographic variables

The demographic and clinical characteristics of the three groups are summarized in Table 1. No significant differences were found among the three groups for age, sex distribution and years of education. No difference was observed between the two BP groups for illness duration, but severity of symptoms measured by HDRS and YMRS were significantly higher in BP II than BP I (Table 1; HDRS: t = 36.91, *P *< 0.001; YMRS: t = 17.22, *P *< 0.001).

**Table 1:** Demographic and clinical characteristics of the three groups

	Control, mean ± SD (N = 20)	Bipolar disorder, mean ± SD		Analysis	
			
		BP I (N = 22)	BP II (N = 29)	**F/χ**^**2**^	*P *value
Age	34.00 ± 12.34	34.05 ± 11.91	34.41 ± 12.19	0.009	0.991
HDRS	-	4.36 ± 2.73	5.90 ± 2.88	36.91	<0.001
YMRS	-	1.86 ± 2.55	3.76 ± 2.66	17.22	<0.001
Illness duration	-	10.40 ± 8.80	11.83 ± 11.78	-0.42	0.676
Educational level	14.65 ± 2.35	13.05 ± 2.99	14.45 ± 3.09	2.067	0.134
Male, N (%)	8 (40.0%)	9 (40.9%)	15 (51.7%)	0.88	0.644

BP = bipolar disorder; HDRS = Hamilton Depression Rating Scale; YMRS = Young Mania Rating Scale.

After using Pearson correlations to examine the relationships among all variables of sustained attention and clinical characteristics, no significant relationships were observed between CPT-II performance and clinical characteristics. Nevertheless, a significant and negative relation was shown between years of education and omission errors in patients with BP I and BP II (*r = *-0.320, *P *< 0.01; Table 2).

**Table 2:** Pearson correlation of demographic characteristics and performance on continuous performance test (CPT) in patients with bipolar disorder (BP) types I and II

	HDRS	YMRS	Age	Years of education
Omission error	0.119	-0.051	-0.149	-0.320**
Commission error	0.118	0.128	-0.157	0.046
Detection	-0.126	-0.150	0.102	-0.001
Hit RT	0.122	-0.013	0.198	-0.191

**P < 0.01.

HDRS = Hamilton Depression Rating Scale; RT = reaction time; YMRS = Young Mania Rating Scale.

### Sustained attention variables (CPT performance)

As shown in Table 3, the hit RT of BP I patients was significantly slower than those of BP II and healthy controls (F = 7.648, *P *= 0.001). The HRT SE of BP II patients and healthy controls were significantly smaller than those with BP I (F = 5.252, *P *= 0.008). After controlling for RT, age, years of education and symptoms severity, MANCOVA analysis revealed significantly increased commission errors (F = 6.182, *P *= 0.004) in patients of BP I than those with BP II and controls. In contrast, on target detection (*d*'), BP II patients and controls scored significantly higher than BP I patients (F = 6.313, *P *= 0.003). No significant difference was found among the three groups on omission errors (F = 0.313, *P *= 0.733) (Table 3).

**Table 3:** Between-group differences for sustained attention measures

	Bipolar disorder (BP), mean ± SD		Control, (N = 20)	Analysis		Bonferroni *post hoc *test
	BP I (N = 22)	BP II (N = 29)	Mean ± SD	**F**_**(2,68)**_	*P *value	
Hit RT^a^			318.63 ± 16.71	7.648	0.001	A > B, C
HRT SE^a^	5.159 ± 0.267	4.070 ± 0.282	4.169 ± 0.383	5.252	0.008	A > B, C
Omission errors^b^	1.764 ± 0.552	2.067 ± 0.543	1.212 ± 0.75	0.313	0.733	
Commission errors^b^	19.490 ± 1.374	14.142 ± 1.351	12.405 ± 1.87	6.182	0.004	A > B, C
*d*'^b^	40.732 ± 7.779	73.825 ± 7.652	77.799 ± 10.57	6.313	0.003	B, C > A

A = BP I; B = BP II; C = control.

^a^Controlling for HDRS, YMRS, educational level and age (MANCOVA).

^b^Controlling for HDRS, YMRS, educational level, age and hit RT (MANCOVA).

*d*' = target detection; HDRS = Hamilton Depression Rating Scale; Hit RT = hit reaction time; HRT SE = hit RT standard error; YMRS = Young Mania Rating Scale.

As shown in Table 4, in all BP participants, there was a significant positive correlation between hit RT and *d*' (r = 0.649, *P *< 0.01). A significant negative correlation between hit RT and commission errors was also found (r = -0.661, *P *< 0.01).

**Table 4:** Pearson Correlation of indexes of performance on continuous performance test (CPT) in patients with bipolar disorder (BP) types I and II

	Omission error	Commission error	Detection
Omission error			
Commission error	0.033		
Detection	0.026	-0.884**	
Hit RT	0.168	-0.661**	0.649**

**P < 0.01.

## Discussion

The present study revealed that although BP II patients presented a higher severity for mood symptoms than BP I, the latter showed a slower hit RT, a greater RT standard error, more commission errors and a lower *d' *than BP II and healthy controls. However, there was no significant difference among BP I, BP II and healthy controls on omission errors. Integrating these findings, it was observed that BP I patients performed worse than BP II and healthy controls on the CPT-II, had more impairments in sustained attention (a significant lower *d*', slower hit RT, and greater RT standard error) and more attentional impulsivity (more commission errors) than those of BP II and healthy controls. Our finding contradict those of Najt *et al*. [39], which illustrated that BP II had longer hit RT than BP I, although only five BP II patients were recruited in their study.

When accuracy is less than perfect, RT covaries with the error rate [40,41]. However, most previous studies that have measured sustained attention among psychiatric disorders have tended to neglect reporting RT [42] and quote the trade-off effect, sacrificing speed for accuracy, as indicated by Lachman *et al*. [38].

Our findings of commission errors in patients with BP I or BP II contradicted that of previous study results [15]. However, the task (go/no-go task) used in the previous study was different from ours, and the authors centralized the commission error as the only index used to measure attentional impulsivity regardless of the trade-off effect, so that hit RT was not incorporated into the study. The present study accepted the concept of attentional impulsivity as mentioned in the study by Swann *et al*. [43], and incorporated both hit RT and commission errors as indexes of attentional impulsivity. As a result, we demonstrated that BP I patients had higher attentional impulsiveness than BP II patients.

No differences in omission errors between BP I and BP II were found in this study. Our results suggest omission errors to be negatively associated with years of education (*r = *-0.320, *P *< 0.01) (Table 2). The possible reason for the lack of difference in omission errors between BP I and BP II might be due to a ceiling effect where the simplicity of the task made for more successful attempts, as no significant difference was found between the two groups in years of education.

### Relations among symptoms, demographic variables and performance on CPT-II

Previous studies indicated that euthymic BP patients also demonstrated impairments in attentional performance [44,45], which allowed us to investigate the correlations between symptoms and CPT performance. In the present study, the symptom rating scores on HDRS and YMRS of BP patients were both 10 or less. No significant correlation existed between the symptoms rated by HDRS or YMRS and CPT-II performance. Our finding was consistent with previous studies that reported no significant correlations between CPT-II performance and the score on the YMRS in manic patients [28,45], or on HDRS in remitted patients [28,46].

Previous reports had shown that age and duration of education did not affect CPT-II performance [28,46]. In contrast, our study found a significant correlation between years of education and CPT-II performance (Table 2). Moreover, omission errors on the CPT-II are suggested to be influenced by age [47]. Therefore, in the statistical analysis, we tried to control for the influence of years of education and age when determining the differences in CPT-II performance between BP I patients and BP II patients. An explanation for this discrepancy might be that it is due to the result of a smaller sample size in the previous study [46]. A significant and negative relation was shown between years of education and omission errors in patients with BP I and BP II (*r = *-0.320, *P *< 0.01) (Table 2).

### Right prefrontal cortex (PFC) and sustained attention measured by CPT-II

Functional neuroimaging studies in healthy volunteers have reported right-lateralized activation in the PFC during continuous performance tests [48,49]. Human lesion evidence also supported that the right PFC was critically involved in sustained attention [50]. The deficit in sustained attention may provide some insight into the neurobiological processes involved in bipolar illness. Accordingly, the different levels of deficit in sustained attention among BP I, BP II and healthy controls demonstrated in our study may suggest possible impairments in the right PFC among BP I patients as compared to BP II patients and healthy controls. This would require further brain imaging studies and other neuropsychological testing to examine the relationship.

### Limitations

A longitudinal follow-up study might provide more information on whether the difference of sustained attention deficit between BP I and BP II is a premorbid issue or if actual progress is related to mood swings during the course of the illness. Additionally, a larger sample size might have illustrated clearer differences between the three groups.

Most of the patients in the present study were on medication. However, no evidence indicated any relationship between medication and CPT-II performance. While a drug-free or drug-washout cohort would be desirable, in clinically severe BP patients the medication is necessary and unavoidable. Remitted patients are needed to make sure the performance on CPT-II was not affected by the medication and severity of symptoms.

To our knowledge, limited studies have focused on the CPT-II performance of BP II patients especially during the interepisode state. This study provided the functional performance of BP II in sustained attention and attentional impulsivity, and revealed differences between BP I and BP II on CPT-II performance. We made comparisons among BP I, BP II and healthy controls on CPT-II performance while controlling for reaction time, which might have confounded the results. In order to prevent the effect of hospitalization, which may influence CPT-II performance, no inpatients were recruited in the present study, reducing the possibility of excess medication or chronicity that may affect CPT-II performance.

## Conclusions

In summary, the present study revealed that BP I patients performed worse than BP II patients on CPT-II performance (slower hit RT and greater hit RT standard error with significantly more commission errors and worse *d' *in patients with BP I). BP I patients had poorer performance in sustained attention and a higher tendency of attentional impulsivity than BP II patients.

Further studies using brain imaging techniques are needed to investigate the difference between the two BP subtypes on sustained attention performance. Rehabilitation interventions should take into account potential sustained attention differences between the two bipolar subtypes, especially in regards to its impact on everyday functions.

## Competing interests

The authors declare that they have no competing interests.

## Authors' contributions

C-HK, R-BL, I-HL, T-LY and Y-KY recruited the participants. C-HK, Y-HC conducted the psychological testing. C-HK, R-BL, S-LC, S-HC, C-HC and Y-HC participated in the design of the study and performed the statistical analysis. C-HK, R-BL, S-LC, JY-WW and S-YL participated in study coordination and helped to draft the manuscript. All authors read and approved the final manuscript.
